# The Interplay among PINK1/PARKIN/Dj-1 Network during Mitochondrial Quality Control in Cancer Biology: Protein Interaction Analysis

**DOI:** 10.3390/cells7100154

**Published:** 2018-09-29

**Authors:** Celia Salazar, Paula Ruiz-Hincapie, Lina María Ruiz

**Affiliations:** 1 Instituto de Investigaciones Biomédicas, Universidad Autónoma de Chile, Santiago 8910060, Chile; celia.salazar@uautonoma.cl; 2 School of Engineering and Technology, University of Hertfordshire, Hatfield AL 10 9AB, UK; p.ruiz-hincapie@herts.ac.uk

**Keywords:** mitochondrial quality control, oxidative stress, PARKIN, PINK1, DJ-1, cancer biology, protein-protein interactions

## Abstract

PARKIN (E3 ubiquitin ligase *PARK2*), PINK1 (PTEN induced kinase 1) and DJ-1 (*PARK7*) are proteins involved in autosomal recessive parkinsonism, and carcinogenic processes. In damaged mitochondria, PINK1’s importing into the inner mitochondrial membrane is prevented, PARKIN presents a partial mitochondrial localization at the outer mitochondrial membrane and DJ-1 relocates to mitochondria when oxidative stress increases. Depletion of these proteins result in abnormal mitochondrial morphology. PINK1, PARKIN, and DJ-1 participate in mitochondrial remodeling and actively regulate mitochondrial quality control. In this review, we highlight that PARKIN, PINK1, and DJ-1 should be regarded as having an important role in Cancer Biology. The STRING database and Gene Ontology (GO) enrichment analysis were performed to consolidate knowledge of well-known protein interactions for PINK1, PARKIN, and DJ-1 and envisage new ones. The enrichment analysis of KEGG pathways showed that the PINK1/PARKIN/DJ-1 network resulted in Parkinson disease as the main feature, while the protein DJ-1 showed enrichment in prostate cancer and p53 signaling pathway. Some predicted transcription factors regulating *PINK1*, *PARK2* (PARKIN) and *PARK7* (DJ-1) gene expression are related to cell cycle control. We can therefore suggest that the interplay among PINK1/PARKIN/DJ-1 network during mitochondrial quality control in cancer biology may occur at the transcriptional level. Further analysis, like a systems biology approach, will be helpful in the understanding of PINK1/PARKIN/DJ-1 network.

Mitochondria are fundamental in numerous cellular functions. The oxidation of carbon sources, the synthesis of adenosine triphosphate, ATP, the generation of reactive oxygen species (ROS), apoptosis, calcium homeostasis, and heme synthesis, among other primordial processes, depend on them. In this same way, mitochondrial dysfunctions are described as the etiological origin of numerous pathologies in mammals such as neurodegenerative diseases, cancer, diabetes, and anemia. One of the main reasons for mitochondrial dysfunction is oxidative stress. Among the damages suffered by the cells are the modification of lipids, alteration of nucleic acids, and damage to proteins. In this review, we will present the most relevant aspects of oxidative stress in mitochondrial biology and the mechanisms of quality control of mitochondria where PARKIN, PINK1, and DJ-1 proteins are involved. In doing so, we try to understand how is the interaction of the network PINK1/PARKIN/DJ-1 during the loss of homeostasis in quality control and how this is associated with carcinogenesis.

## 1. Biochemistry of Reactive Oxygen and Nitrogen Species (ROS/RNS)

Diatomic oxygen (O_2_) has a high redox potential making it an oxidizing agent capable of accepting electrons from reduced substrates [1]. The reactive oxygen species (ROS) generated in the mitochondrial network primarily originate from O_2_^•−^, and from its acidic conjugate hydroperoxyl radical HO_2_^•^ which is soluble in membranes [2,3]. However, hydroxyl (OH^•^), carbonate (CO_3_^•−^), peroxyl (RO_2_^•^), and alkoxyl (RO^•^) radicals are also potential originators [4]. Mitochondrial O_2_^•−^ production by reduction of O_2_ single electron is thermodynamically preferred, even by relatively oxidizing redox couples, due to being an event that occurs within the proteins’ redox-active prosthetic groups. However, mitochondrial O_2_^•−^ production is also achieved when electron carriers such as CoQH2 (NADH, NADPH, Glutathione) are bound to proteins hence, driving the kinetic factors determine the O_2_^•−^ production [5,6]. Additionally, transition metals like Copper (Cu), Iron (Fe) and Zinc (Zn) destabilize spin states at the outer orbital electrons in O_2_ and generate ROS such as O_2_^•−^, hydrogen peroxide (H_2_O_2_) and hydroxyl radical (OH^•^) [1]. By contrast, Fenton reactions are accountable for the most reactive hydroxyl radical OH^•^ originating from H_2_O_2_ (Table 1) [4,7]. O_2_^•−^ and H_2_O_2_ can be generated from major sites at high rates both in the mitochondrial matrix and in the cytosolic side of the mitochondrial inner membrane. However, mitochondrial and cytosolic scavenging of O_2_^•−^ and H_2_O_2_ are also very powerful, for instance, four sets of dynamic balance between production and consumption rates regulate the levels of O_2_^•−^ and H_2_O_2_ [6].

Under hypoxic conditions, the mitochondrial respiratory chain also produces nitric oxide (NO^•^) that in turn can lead to the production of further reactive nitrogen species (RNS) [1]. Similarly, NO^•^ can react with O_2_^•−^ to form strong oxidant peroxynitrite (ONOO^−^) [1]. It is therefore reasonable to suggest that the best-understood function of mitochondrial ROS and NO^•^ is their role in oxygen sensing [1], whereby high oxygen levels would favor O_2_^•−^, while low oxygen levels would favor NO^•^; however, intermediate oxygen levels would prefer ONOO^−^ [1]. Nitrosative and oxidate stresses have been identified in aging and several diseases, including neurodegenerative diseases and cancer. Cancer cells are skilled at upregulating survival signaling pathways and must respond to the high levels of NO and ROS, which are byproducts of their growth.

## 2. How are Free Radicals Produced in the Mitochondria?

A mitochondrion is characterized by a complex double membrane structure housing cristae (formation of cavities and folds within the inner mitochondrial (IMM) membrane whose longitudinal axis is at right angles to the inner boundary layer) and by continuous tubules of the mitochondrial reticulum network [6,8,9]. The respiratory chain is usually considered one of the primary sources of ROS. The oxidative phosphorylation (OXPHOS) housed in the IMM, plays a crucial role in energy production and the generation of ROS [10]. The OXPHOS system comprises of the electron transport chain (ETC) formed by four respiratory complexes (Complex I, NADH dehydrogenase; Complex II, succinate dehydrogenase; Complex III, ubiquinol cytochrome c reductase; and Complex IV, cytochrome c oxidase) plus the mobile electron carriers (e.g., cytochrome c and coenzyme Q) [11]. The OXPHOS machinery is organized into higher-order complexes called respiratory supercomplexes or respirosomes, which are formed by the agglomeration of complexes I, III, and IV in the membrane of mitochondria in different configurations. These supercomplexes or respirosomes form functional units in the ETC through which electrons are channeled [12,13].

Within the Krebs cycle, ETC and β-oxidation in mammalian mitochondria a minimum of ten different sites for O_2_^•−^/H_2_O_2_ production have so far been identified. Complex I and complex III are the most critical mitochondrial O_2_^•−^ producers but other sites have also been acknowledged, including pyruvate dehydrogenase, glycerol phosphate dehydrogenase, and α-ketoglutarate dehydrogenase and ETF-CoQ reductase [14]. The factors managing mitochondrial ROS levels are associated to their own generation and removal rate, while the regulation of the concentration of redox species responsible for electron leaking and ROS generation is mediated by the redox potential of the NAD+/NADH coupling degree and the proton-motive force (Δp) [7,15]. In turn, these events are regulated by the redox supply to the respiratory chain achieved via the number of couplings made and constraints encountered during the electron transfer [7,15]. Superoxide Dismutase (MnSOD) in the mitochondrial matrix catalyzes the dismutation of O_2_^•−^ very rapidly into non-radical H_2_O_2_ which is regarded as a stable species as well as a primordial mediator in redox regulations by implementing relay or diffusion through the redox buffer system [16,17]. It is also worth mentioning that the Superoxide Dismutase (CuZnSOD) in the mitochondrial intermembrane space and cytosol dismutes O_2_^•−^ to H_2_O_2_ [16,17].

In mammals, complex I is the entry point for electrons from NADH into the respiratory chain. It is also a ~1 MDa complex comprising 45 proteins, seven of which are mitochondrially encoded subunits [18,19]. Complex I produce large amounts of O_2_^•−^ by two mechanisms: (1) high mitochondrial matrix NADH/NAD+ ratio, leading to a reduced flavin mononucleotide site on complex I, and (2) when electron donation to the coenzyme Q (CoQ) pool is accompanied by a high Δp and a lack of ATP synthesis leading to a reversal in the electron transfer (Re-t) [5]. So far, research findings seems to point out at Re-t mediated O_2_^•−^ production rate as being the highest ever found in mitochondria [20]. Under mitochondrial physiological conditions, complex III is yet another originator of O_2_^•−^ however, this source is minor compared that achieved by complex I [17,21]. Nevertheless, in conditions where complex I is either absent or low then O_2_^•−^ production by complex III assumes a more significant role [5]. For mitochondria that are either actively synthesizing ATP or utilizing Δp for other functions (state 3), both a low Δp and an oxidized NADH pool prevent O_2_^•−^ production by Re-t and significantly decrease it at the flavine mononucleotide of complex I. Although, O_2_^•−^ production rate is almost minimal in state 3, this particular state might be the driver of the highest biological grade and be accountable for the cumulative mitochondrial oxidative damage and the enormous tissue damage as found in the absence of MnSOD. Additionally, at low oxygen concentration complex IV (CIV) produces NO^•^ from nitrite (NO_2_^−^) [22]. Inhibition of complex IV may facilitate ROS production by complexes I or III [10].

## 3. Mitochondrial Quality Control Systems

Within the human mitochondrial matrix, 495 proteins have thus far been identified [23]. Many of the coordinated protein quality control systems are localized in the mitochondrial matrix. These control systems encompass the Lon, ClpXP, and m-AAA proteases, which are proteases of AAA+ conserved superfamily. The latter have been suggested to be highly involved in important events of the mitochondrial proteins, found in the matrix and the inner membrane, including degradation, processing, and supervision of the proteins assembly. The inner membrane is in turn involved in oxidative phosphorylation, mitochondrial protein synthesis, mitochondrial network dynamics, and nucleoid function. The quality control proteases are in turn upregulated by a variety of mitochondrial stressors, including oxidative stress, unfolded protein stress and imbalances in the assembly of respiratory complexes [24].

The mitochondria have highly developed networks that are kept in good working order by the contrasting events of fusion and fission [25,26]. Each fusion event requires two reactions: an initial one triggered at the outer membrane followed by reaction of the inner membrane [27,28]. In mammals, these reactions are catalyzed by dedicated GTP-ases (Mfn1, Mfn2, and OPA1) [29,30]. By contrast, mitochondrial fission is a separation event in which a mitochondrion is split into two organelles [31]. In mammals, this event is moderated by Drp1, a cytosolic dynamin-like protein that constricts the mitochondrial membrane and promotes mitochondrial fragmentation (fission) [32,33]. Drp1 binds to mitochondrial fission protein 1 (Fis1) at the mitochondrial outer membrane. Preservation of a fit and functional mitochondrial network is vital in the adequate response to physiological changes and stressful environments. Unfortunately, due to exposure to high levels of ROS in their role in energy production, mitochondria is a damage-prone structure [6]. Mitochondria have developed elaborate mechanisms of quality control regulated by two opposing events: the removal of damaged organelles or their components and mitochondrial biogenesis to ensure that the proper quantity of functional mitochondria is available to counter the cellular stresses. The elimination of whole mitochondria is accomplished by a selective form of autophagy called mitophagy [34,35]. Fusion, fission, and mitophagy are important moderators of mitochondrial turnover and homeostasis; the combination of these processes shape the cell’s survival decisions [36,37].

In this review, we focus on the regulatory processes of proteins PINK1, PARKIN, and DJ-1 specifically during mitochondrial quality control due to its importance in cancer biology. PARKIN (Parkin RBR E3 Ubiquitin Protein Ligase, *PARK2* gene), PINK1 (PTEN Induced Putative Kinase 1, *PINK1* gene), and DJ-1 (Parkinsonism Associated Deglycase, *PARK7* gene) are proteins involved in autosomal recessive parkinsonism [38,39,40,41], and modulators of carcinogenic processes [42,43,44,45,46,47].

In damaged mitochondria, PINK1’s importing into the inner mitochondrial membrane is prevented, PARKIN presents a partial mitochondrial localization at the outer mitochondrial membrane and DJ-1 relocates to mitochondria when oxidative stress increases [48,49,50,51,52,53,54,55,56]. These proteins actively regulate mitochondrial quality control [48,51,52,56,57,58] and are involved in mitochondrial remodeling [53,59,60,61] hence, their depletion or even loss is a cause of changes in the mitochondria’s morphology [53,55]. The interaction of PINK1 and PARKIN regulates mitochondrial dynamics [62,63,64,65,66,67] and also mediates mitochondrial quality control [51,56,59,60,61,62]. They are proteins that mediate mitophagy of malfunctioning mitochondria when they cooperate with each other [68,69]. Interestingly, PINK1 and PARKIN’s reciprocity aids the regulation of mitochondrial morphology via mitochondrial fission and fusion, and this in turn has an impact on mitochondrial quality control [59,61,64,67]. Only recently, DJ-1 was found to have a role in mitochondrial quality control [57,58,70]; it brings a protective function by aiding mitochondrial homeostasis or by responding to oxidative stresses [71,72].

### 3.1. PINK1′s Role in Mitochondrial Quality Control

*PINK1* gene encodes PTEN Induced Putative Kinase 1, a serine/threonine kinase homologous to kinases that are moderated by calcium and/or calmodium [40]. The exogenous expression of PTEN (Phosphatase and Tensin Homolog), a primary tumor suppressor, upregulates PINK1 [73]. PINK1 has been found in mitochondria in all cell types from rat and human brain tissue [74]. PINK1 protein spans the outer mitochondrial membrane (OMM). It has a topology in which the kinase domain in the C-terminal faces the cytoplasm while the N-terminal tail contained inside the mitochondria, near the canonical N-terminal mitochondrial targeting signal (MTS), is located in the transmembrane (TM) domain [55]. This domain is necessary for PINK to attach to the mitochondrial membrane with its kinase domain facing the cytoplasm [55]. PINK also displays neuroprotective roles including stabilization of the mitochondrial membrane potential; halting the release of apoptogenic factors such as cytochrome c; inhibiting O_2_^•−^ production [59,64,75,76,77]. The role of PINK1 in mitochondrial dynamics was first identified in studies done in a *Drosophila* model in which lack of PINK1 resulted in adverse conditions for mitochondria’s post-mitotic tissues [59].

The study of silencing *PINK1* gene expression in different models, including cultured mammalian cells, *Drosophila*, mouse and primary fibroblasts in a human subject with mutated PINK1 revealed a highly abnormal morphology presenting loss of membrane potential and increased oxidative stress; thus, associating PINK1 with mitochondrial homeostasis control [61,64,67,70,77,78,79,80,81]. Surprisingly, these alterations in mitochondrial physiology were found to be revoked by either a fluctuating or steady expression of PARKIN suggesting that PARKIN is a downstream effector of PINK1 in the regulation of mitochondrial homeostasis [64]. Not only was the induction of mitochondrial fragmentation and autophagy of PINK1 knockdown rescued with PARKIN, its overexpression caused an augmented autophagic and mitophagy response [62]. The latter was also reported recently in a study focusing on PARKIN’s role in autophagic cleansing of depolarized mitochondria [51,56].

PINK1 supports mitochondrial membrane potential; oxidative phosphorylation [82,83,84] complex I activity [84]; ROS and Ca^2+^ balance [85]; protects from mitochondrial stress [62,63,83,86], regulates mitochondrial dynamics [61]; key regulator of mitophagy [87,88]. It is also involved in the upkeep of healthy mitochondrial networks which in turn allows for the migration of mitochondria to synapses via Miro and Milton, kinesin adaptor-like proteins [89].

The appearance of unfolded proteins in the matrix acts as a sensor that promotes PINK1 buildup in mitochondria with healthy bioenergetics; thus, causing PARKIN migration and mitophagy; eventually, reducing the amount of unfolded protein. Likewise, silencing of the LONP1 protease enhances PINK1 accumulation [90]. While PINK build up is the precursor of mitophagy as a quality control process, PARKIN assumes a cleansing role by removing defective mitochondria via autophagy [51,52,56,91].

### 3.2. PARKIN’s Role in Mitochondrial Quality Control.

PARKIN is a protein, the after encoded by PARK2 gene, that acts as a E3 ubiquitin ligase, it is part of the ubiquitin-proteasome system [92,93]. It is typically found in the cytoplasma of most cells but would translocate to mitochondria when the organelle is damaged [94,95].

A variety of human cancers present alterations in chromosome 6q25–27 [92,96]; PARKIN is a potential tumor suppressor gene for that chromosome [97]. Thus, PARKIN mutations resulting in changes in this chromosome are potentially a risk factor for neurodegeneration and cancer.

The phosphorylation of serine 65 (S65) amino acid residue in the Ubl domain in PARKIN mediated by PINK1 is critical for its recruitment to the mitochondrial outer membrane [94,98]. The same phosphorylation also generates a type of phospo-ubiquitin (pUb) [94,99,100] that triggers morphologic changes leading to the release of the UbI domain. The latter allows for PARKIN’s center to be phosphorylated by PINK1 [101,102,103]. Mutations of the S65 domain in PARKIN produces physiologically inactive proteins that alter the mitochondria’s ability to recruit PARKIN; hence, producing a misbalance in cleansing activities such as mitophagy [104]. Thus, the protein PARKIN presents a basal auto-inhibited state, for its enzymatic activity in mitophagy requires the activation through the S65 phosphorylation, although it is not clear if the S65 phosphorylation step activation is necessary for PARKIN mediated ubiquitination in non-mitophagy events [105].

The S-nitrosylation of PARKIN inhibits its neuroprotective ability as found in the brains of patients with PD [106]. This modification arises, after its reaction with nitric oxide (NO) and as a consequence producing SNO-PARKIN [107,108]. E3 ubiquitin ligase is then briefly stimulated before it becomes wholly dysfunctional, initiating a atypical protein production [18,19].

PARKIN is involved in mitochondrial homeostasis however; it is not active in the cytosol. PARKIN’s translocation to mitochondria by a decrease in mitochondrial membrane potential depends on PINK1 expression [54]. In ROS-driven processes, PARKIN translocates to mitochondria [109] of primary neurons; by contrast, it translocates to the uncoupled mitochondria of tumor cell lines [110], and it participates in the clearance of damaged mitochondria under PINK1’s influence [51,54,56,91,111,112]. The importance of the PINK1/PARKIN interactions in mitochondrial quality control in neurodegeneration has been elegantly reviewed by Cummins and Götz (2018) [113]. The PINK1-PARKIN pathway mediates the mitophagy of dysfunctional mitochondria in distal neuronal axons [114]. The detection of phosphorylated ubiquitin in the brains of Mutator mice that accumulates dysfunctional mitochondria indicates the activation of PINK1-Parkin [115]. The loss of Parkin in the Mutator mice cause degeneration of dopaminergic neurons and motor deficit. This work demonstrates a role of endogenous Parkin in the preservation of dopaminergic neurons [115].

### 3.3. DJ-1 in Mitochondrial Quality Control

The locus of the *PARK7* gene, found on the short arm of chromosome 1 (1p36.23); encodes the DJ-1 protein. The latter is a multifunctional protein, initially identified as an oncogene [116] due to its increased presence in carcinomas such as melanoma and breast cancer [117], well preserved in species yet highly standing in most cells and tissues in the body [118]. DJ-1 is participating in cell survival [119], apoptosis [120], transcriptional regulation [121,122], and oxidative stress mechanisms [70,121,123], among others. DJ-1 localizes mainly in the cytoplasm at the basal condition; but is also found in the nucleus and mitochondria [124]. During oxidative stress, DJ-1 is translocated to the mitochondria for its protective role [49,121]. During the S-phase of the cell cycle, with the aid of growth factors and oxidative stress, DJ-1 translocates to the nucleus [124,125].

Early-onset of PD results after homozygous loss-of-function mutations in the *PARK7* gene [38]. Several studies have provided strong evidence that DJ-1 safeguards neurons against cell death due to oxidative stresses [72,126]. This protective function is either achieved by gathering antioxidants or acting as a redox sensor [71,72,127,128].

DJ-1 deficiency in *DJ-1^−/−^* mice results in elevated ROS levels and a fragmented mitochondrial phenotype in primary cortical neurons; mouse embryonic fibroblasts (MEFs) and brain; striatum [129]. DJ-1-dependent mitochondrial fragmentation was related to a decreased in MFN1 fusion protein levels, and mitochondrial fusion rates [129]. This phenotype can be rescued by PINK1 and PARKIN resulting in increased autophagic activity [66,129]. DJ-1 integrates into the classical PINK1/PARKIN pathway by modulating PARKIN translocation/mitophagy and responding to PINK1/PARKIN by increasing the level of mitochondria activity during oxidative stress [66,109].

## 4. Relation of PINK-1/PARKIN/DJ-1 Network in Cancer Biology

Cancer is characterized by extensive cellular proliferation, while premature cell death marks neurodegeneration. Both processes share biological pathways differentially regulated [130,131]. Remarkably, patients with PD have presented low cancer incidence [132,133,134,135,136,137,138,139]. The evidence exposes a different pattern of cancer in certain neurological conditions suggesting that neurological diseases may prevent cancer [132]. Unfortunately, comorbidity (presence of new diseases about an index disease) in medical health investigation and practice is comparatively low with that of individual diseases [140,141]. Many factors can explain this duality including biological ones, as opposed to genes and pathways, as well as inorganic ones, such as behaviors, characteristic patterns or effects of medication. Past observational studies have shown the “comorbidity of cancer and disorders of the central nervous system (CNS)” [142], while newer studies suggest a lower incidence of some types of cancer in certain pre-existing CNS disorders [133,143]. The latter has been termed “inverse cancer comorbidity” and has been reported in individuals with Schizophrenia and PD, mainly for colorectal and prostate cancers [144]. Contradictorily, melanoma and breast cancer display high incidence in patients with PD [145]; however, this could be linked to the presence of malfunctioning autophagy in the two conditions [116,146,147].

Defects in autophagy have been linked to neurodegenerative diseases [148,149,150,151]. Some studies have shown that it inhibits tumor initiation, but in the presence of hypoxia and/or metabolic stress this role is reversed and instead supports the survival of existing tumors [152]. Interestingly, in melanoma the autophagy genes Beclin1 and LC3 have been found to be decreased [153], yet melanoma cells show high levels of autophagy [154].

### 4.1. PARKIN Signaling in Cancer Biology

**Figure 1:**
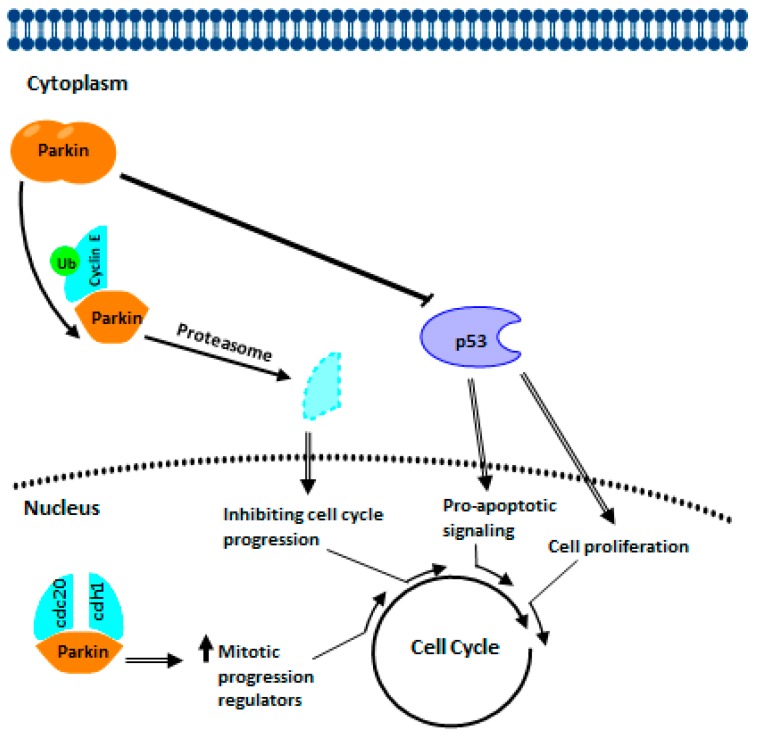
Graphical representation of the interactions of the PARKIN proteins. The directionality and the nature of the interactions (activation or inhibition) are shown as they are believed to exist in a healthy state.

Many types of human cancers downregulate PARKIN expression [155,156]. The *PARK2* gene is a tumor suppressor; hence, it is a target of research in a variety of cancers, while mutations in *PARK2* in somatic cells contribute to oncogenesis [46,93,157]. Remarkably, PARKIN behaves as an “ubiquitin-protein ligase” having cyclin E (G1 cyclin) as one of their well-recognized substrates (Figure 1). The deficiency of PARKIN is associated with the accumulation of these types of cyclins in breast cancer development [156]. Overexpression of PARKIN represses cell growth by degradation of cyclin E mediated by the ubiquitin [157,158]. In colon and brain cancer cells with dysfunctional PARKIN show alterations in the proteolysis of cyclin E [92,159]. Besides, PARKIN regulates mitosis through the interaction with Cdc20 and Cdh1 (Figure 1); these are coactivators of the anaphase-promoting complex/cyclosome [160]. Studies performed by Tay et al. [156] found that restoring PARKIN’s expression in MCF7 breast cancer cells reduce their growth rate and motility. The former is likely due to the cells’ sudden cessation during the G1 phase of the cell cycle.

Interestingly, a microarray analysis of MCF7 cells expressing functional PARKIN exposed an important increment in the expression of cyclin-dependent kinase 6 (CDK6) [161]. PARKIN overexpression in cancer cells results in a significant decrement in their growth rate [92]. Clearly, CDK6 are now well known for downregulating the growth of breast cancer cells, suggesting a potential tumor-suppressing mechanism of PARKIN on breast cancer cells [156]. Additionally, in breast cancer, PARKIN stabilizes microtubules and rises cancer vulnerability to oncologic drugs [162]. In HeLa cells, a human cervical cancer line, the expression of PARKIN modulates caspase activity and survivin protein; these promote susceptibility to TNFα-induced cell death [163]. In the presence of functional PARKIN, via cyclin E downregulation and AKT signaling, glioma cells decrease their proliferation [92]. In other words, activation of the PARKIN pathway is an indicator of “survival prognosis” [92]. Notably, *PARK2* gene expression is often decreased in human cancers, leading to a dysregulated PI3K (phosphatidylinositol 3-kinase) signaling. These outcomes were contingent on the appearance of PTEN, suggesting that loss of PARKIN, directly or indirectly, impaired the tumor suppressor activity of PTEN [164].

### 4.2. PINK1 Signaling in Cancer Biology

**Figure 2:**
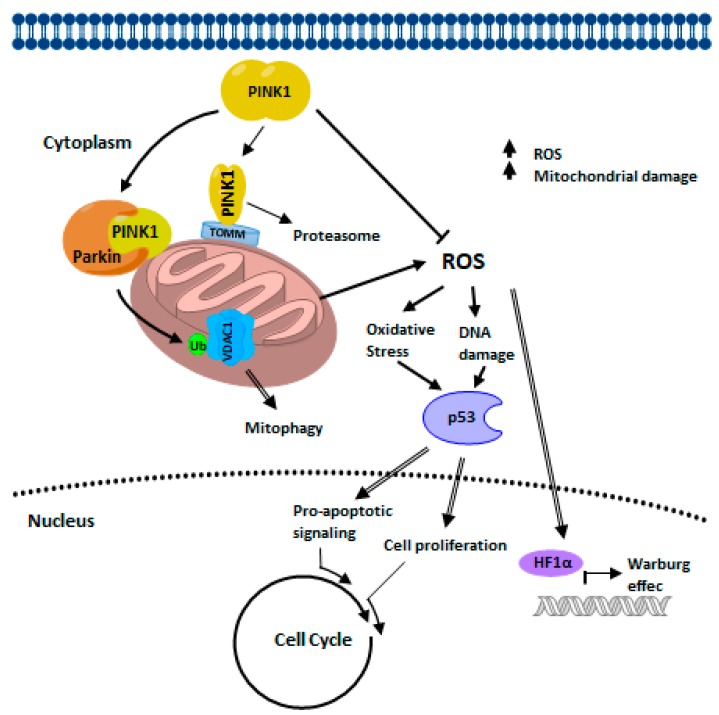
Graphical representation of the interactions of the PINK proteins. The directionality and the nature of the interactions (activation or inhibition) are shown as they are believed to exist in a healthy state.

PINK’s role in cancer cell biology is not well understood. The Figure 2 show a graphical representation of the interactions of the PINK1 protein, as they are believed to exist in a healthy state. The majority of the work done so far has been related to PINK’s role in mitochondrial function in PD however, many of the experiments in those studies were carried out in cancer cell lines [54,56,67,91,110,165,166]. Significant findings in this field of research suggest that PINK’s absence in cancer cells promotes fission which in turn results in higher number of fragmented mitochondria [45,62,63]. It was also found that PINK’s expression was higher in mice cancer cell lines that have the higher metastatic potential [167]. Notably, PTEN family may participate in multiple distinct cellular functions, and the isoform PTENα is essential for mitochondrial energy metabolism. Cells lacking PTENα show a reduced level of PINK1 expression [168]. Interestingly, the deletion of PINK1 in immortalized mouse embryonic fibroblasts (MEFs) have been found to reduce cancer-associated phenotypes, and this was seen to be reversed by the overexpression of human PINK1. The knockout mice for PINK1 present significant defects in cell cycle progression, this indicates that PINK1 has tumor-promoting properties [169].

High glycolysis and impaired mitochondrial metabolism characterize the metabolic reprogramming of cancer cells. Loss of PINK1 in glioblastoma contributes to the Warburg effect through regulators of aerobic glycolysis [170]. Mainly, in Human brain tumors with poor patient survival deletion of PINK1 in association with regulators of aerobic glycolysis give rise to the Warburg effect [170].

### 4.3. DJ-1 Signaling in Cancer Biology

**Figure 3:**
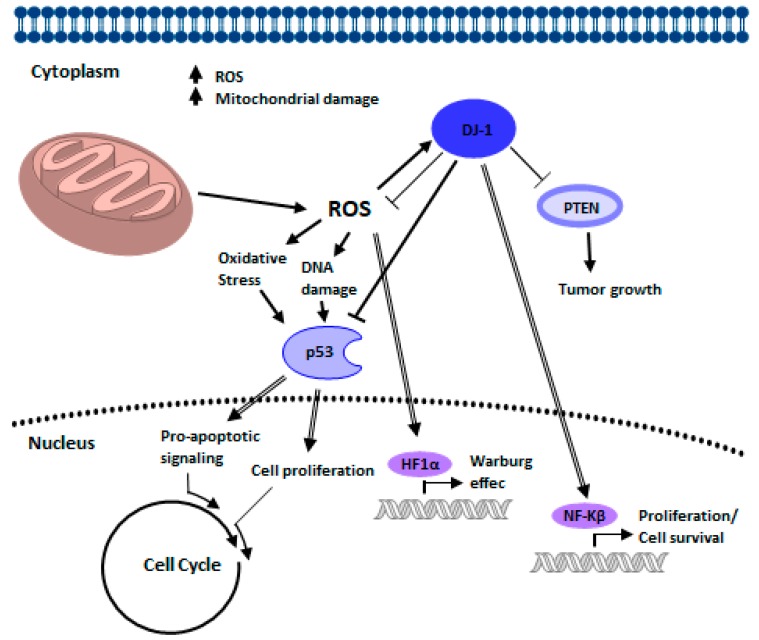
Graphical representation of the interactions of the PINK proteins. The directionality and the nature of the interactions (activation or inhibition) are shown as they are believed to exist in a healthy state.

Curiously, DJ-1 was isolated for the first time in 1997 when studying c-Myc-binding proteins (proto-oncogene) [125], although DJ-1 does not interact directly with c-Myc, the *PARK7* gene in combination with H-Ras transform mice NIH3T3 cells into cancerogenic ones [125]. Some understanding in the relationship between DJ-1 and cancer has been gathered [43,58], DJ-1 is now known to act as a mitogen-dependent oncogene [125,171,172] (Figure 3). Precise interactions between the biochemical function of DJ-1 and its subcellular localization are yet to be elucidated; however, the DJ-1 malfunction has been linked with cancer onset. Various malignant tumor cells, including prostate cancer, non-small cell lung cancer, primary lung cancer, laryngeal cancer, ovarian carcinoma, cervical cancer, and endometrial cancer, show a high DJ-1 expression [38,173,174,175]. DJ-1 plays a role in increasing cell proliferation and metastasis [176,177]. DJ-1 knockdown in cancer cells significantly reduces in vitro cell proliferation and migration, and in vivo tumor growth [47]. DJ-1 promotes oncogenesis mediating the cell survival supported by upregulation of protein kinase B (PKB)/Akt [125,178]. The studies conducted by Lin et al. [179], in surgical medulloblastoma tissue specimens and paired tumor-adjacent tissue specimens, it was observed that tumor cells had a high DJ-1 expression and this in turn was linked to high growth rate and undifferentiated tumors, while metastasis stage tumors and high-risk tumors showed a high p-Akt expression. Also, DJ-1 antagonize the tumor suppressor PTEN, inhibiting *PTEN* gene activity and promoting tumor cells proliferation [178]. The loss or decrease of *PTEN* seems to be a joint event in many types of tumors. Downregulation of PTEN and high expression of DJ-1 correlate with poor prognosis in gastric carcinoma [174]. Moreover, DJ-1 interacts with HER3, this protein plays a crucial role in cell proliferation and survival [180]. Neuregulin (NRG) binding to HER3 induces heterodimerization of HER3 with other EGFR family receptors, resulting in its phosphorylation. Phosphotyrosines of HER3 provide binding sites for PI3K and other HER3 interacting proteins [181,182], which mediate activation of the PI3K/AKT and Ras/Raf/MAPK pathways [183,184,185]. The overexpression of DJ-1 in cancer cells increased the level of HER3 and promoted proliferation and tumor growth [47]. Tumor tissues of breast cancer patients have shown co-expression of HER3 and DJ-1. Thus, high expression of DJ-1 in breast cancer cells predicts elevated HER3 signaling [47]. Furthermore, DJ-1 regulated the transcription factor nuclear factor-κβ (NF-κβ) enhancing its nuclear translocation and cell survival [186]. NF-κβ is critical in cancer development [187,188]. DJ-1 also directly interacts with Cezanne, which networks with NF-κβ reducing its nuclear translocation working as a physiological inhibitor of its transcriptional activity [186].

Remarkably, the striatin family SG2NA protein was first characterized as an autoantigen in a cancer patient and described as an oncogene [189,190]. Like DJ-1, SG2NA was also found to be an oncogene that in cooperation with ras transforms NIH3T3 cells [125]. DJ-1 and Akt are recruited to the plasma membrane and mitochondria by SG2NA, to protect cells from oxidative stress [191]. Interestingly, DJ-1 isoform in serum with isoelectric point (pI) of 6.3 is prevalent in breast cancer patients, suggesting that these proteins can act as potential markers for breast cancer [192]. Intriguingly, studies of multidrug resistance (MDR) in humans originating from chemotherapy in cancer treatment have identified that silencing DJ-1 successfully reverses MDR [193].

## 5. Protein-Protein Interactions Analysis of PINK1/PARKIN/DJ-1 Network

The STRING database, complemented with heuristic methods of association and analysis, was used to consolidate known and predicted protein-protein association for PINK1, PARKIN, and DJ-1. The STRING (search tool for the retrieval of interacting genes) database generates a network of protein interactions from high-throughput experimental data, literature, and predictions based on genomic context analysis [194,195]. Interactions in STRING are derived from five main sources: Genomic Context Predictions, High-throughput Lab Experiments, (Conserved) Co-Expression, Automated Textmining and Previous Knowledge in Databases.

**Figure 4:**
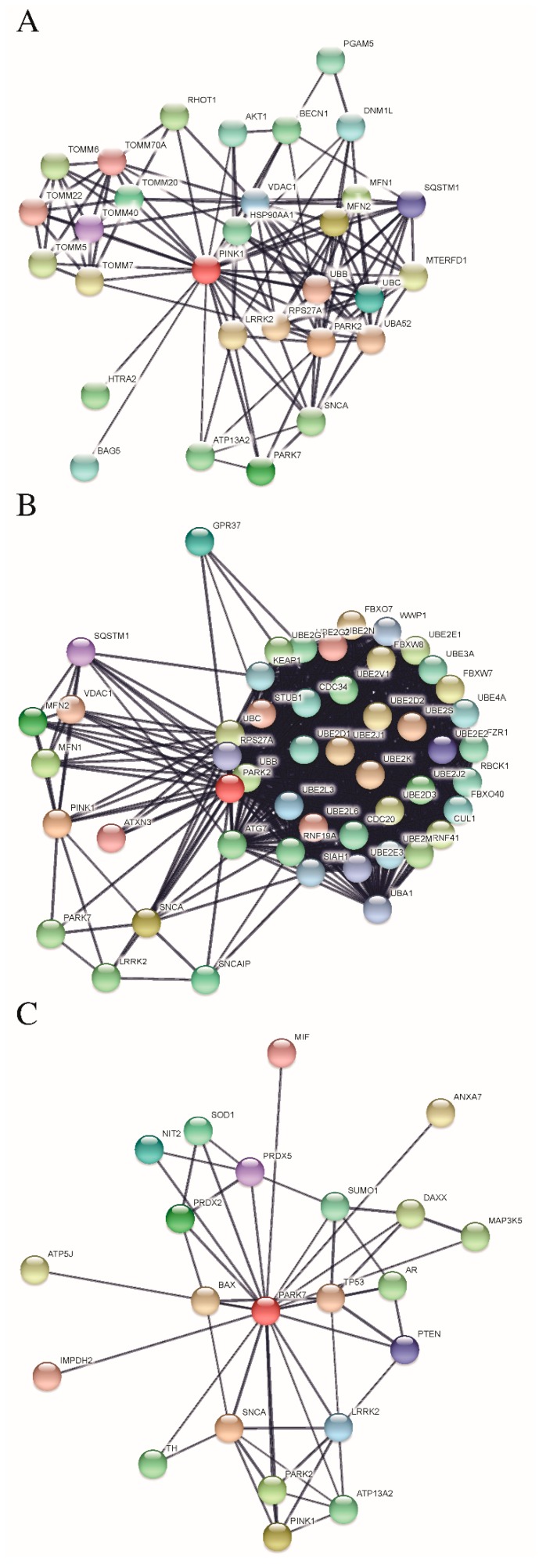
STRING analysis reveals protein interaction networks of the proteins DJ-1 (PARK7) (**A**), PINK1 (**B**) and PARKIN (PARK2) (**C**) with other proteins. The interaction network was created with STRING (Search Tool for the Retrieval of Interacting Genes/Proteins) database version 10.5. A high confidence cutoff of 0.7 was implemented in this work. In the resulting protein association network, proteins are presented as nodes which are connected by lines whose thickness represents the confidence level (0.7–0.9).

**Figure 5:**
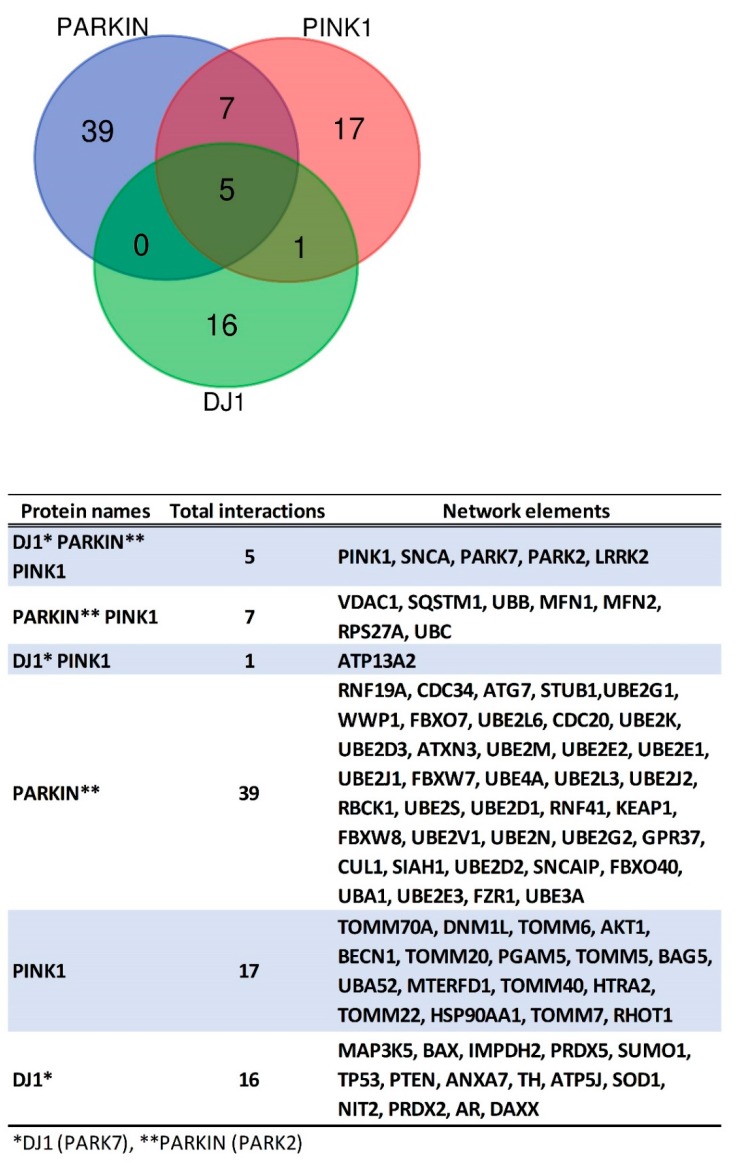
Venn diagram of PINK1/PARKIN/DJ-1 network. This was created with the Bioinformatics and Evolutionary Genomics tool.

The top predicted functional partners of PINK1, PARKIN, and DJ-1 are shown in Figure 4. A Venn diagram, displaying a three-way interaction network, was assembled by implementing the Bioinformatics and Evolutionary Genomics Tool [196]. The three proteins interacted with each other and with SNCA (alpha Synuclein) and LRRK2 (Leucine-rich repeat kinase 2) (Figure 5). On the one hand, the protein SNCA induces fibrillization of microtubule-associated protein TAU while neuronal responsiveness to various apoptotic stimuli was reduced. On the other hand, the protein LRRK2 positively regulates autophagy through a calcium-dependent activation of CaMKK/AMPK signaling pathway. PARKIN and PINK1 are interacting in the network with VDAC1 (Voltage-dependent anion channel 1), SQSTM1 (Sequestosome 1), UBB (Ubiquitin B), MFN1 (Mitofusin 1), MFN2 (Mitofusin 2), RPS27A (Ribosomal protein S27a; Ubiquitin) and UBC (Ubiquitin C). In the case of DJ-1 and PINK1 interactions were established with ATP13A2 (ATPase type 13A2). Figure 5 shows the predicted functional partners of PINK1, PARKIN, and DJ-1. It is important to acknowledge that in the interaction analysis of protein PARKIN maybe included the interactions with the autoinhibited PARKIN and with the activated PARKIN. We trust that all the possible interactions with the autoinhibited or activated PARKIN might have a biological function.

**Figure 6:**
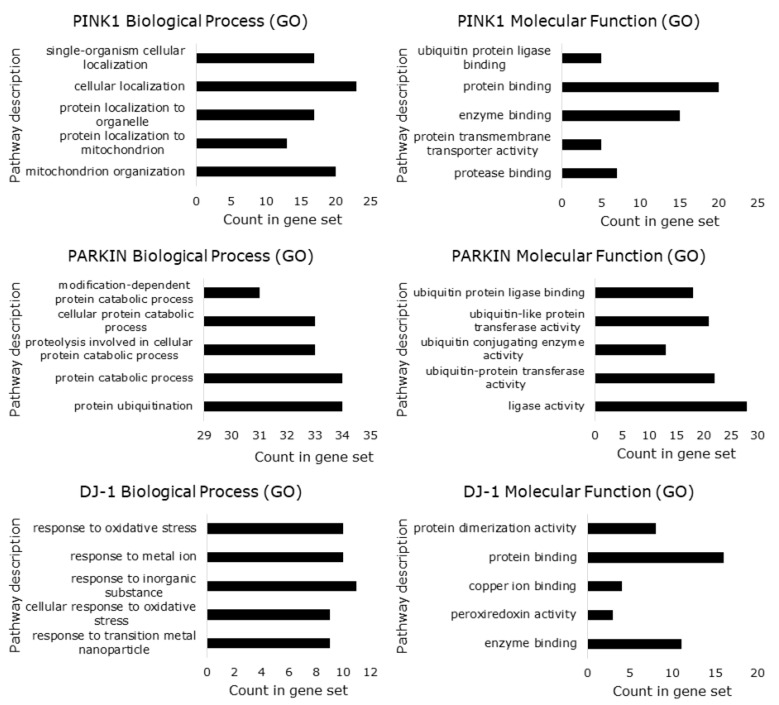
Gene ontology enrichment analysis for biological processes and molecular function in the interaction networks of PINK1, PARKIN, and DJ-1. The numbers on the horizontal axis represent the enrichment score. A high enrichment score related to the genes were found more frequently in the ontology.

**Figure 7:**
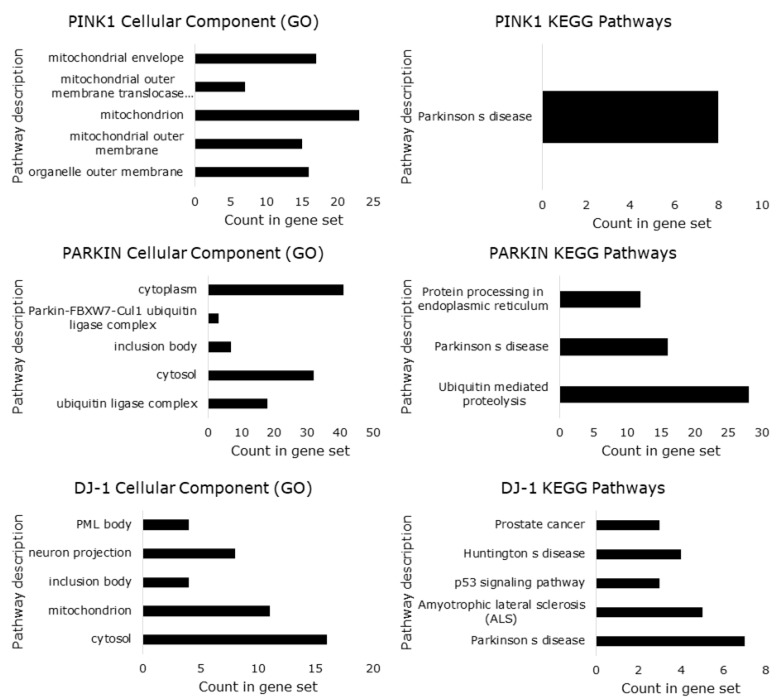
Gene ontology enrichment analysis for cellular component and KEGG pathways in the interaction networks of PINK1, PARKIN, and DJ-1. The numbers on the horizontal axis represent the enrichment score. A high enrichment score related to the genes were found more frequently in the ontology.

A gene ontology (GO) analysis [195] was performed to establish the role of enrichment in biological processes, molecular function, and cellular components in the interaction networks of PINK1, PARKIN, and DJ-1. The enrichment analysis showed that for biological processes in the PINK1 system most of the genes were enriched at a “cellular localization” and at a “mitochondrion organization” level (Figure 6). For the PARKIN network, most of the genes show enrichment in “protein ubiquitination” and “catabolic process” (Figure 6). “Response to an inorganic substance (metal ion)” and “response to oxidative stress” were higher for DJ-1 network (Figure 6). The typical molecular functions were protein binding for PINK1 and DJ-1 network, and ligase activity for PARKIN network (Figure 6). The cellular component analysis showed that most of the genes were higher in mitochondrion for the PINK1 system, cytoplasm for the PARKIN network, and cytosol and mitochondrion for the DJ-1 network (Figure 7). The enrichment analysis of the KEGG pathways showed that the PINK1/PARKIN/DJ-1 network was Parkinson disease (Figure 7). Only the protein DJ-1 showed enrichment in prostate cancer and p53 signaling pathway (Figure 7). This kind of analysis will be useful in the understanding of the interplay among PINK1, PARKIN, and DJ-1 in cancer biology.

## 6. Predicted Transcription Factor Regulating *PINK1, PARK2* and *PARK7* Gene Expression

**Figure 8:**
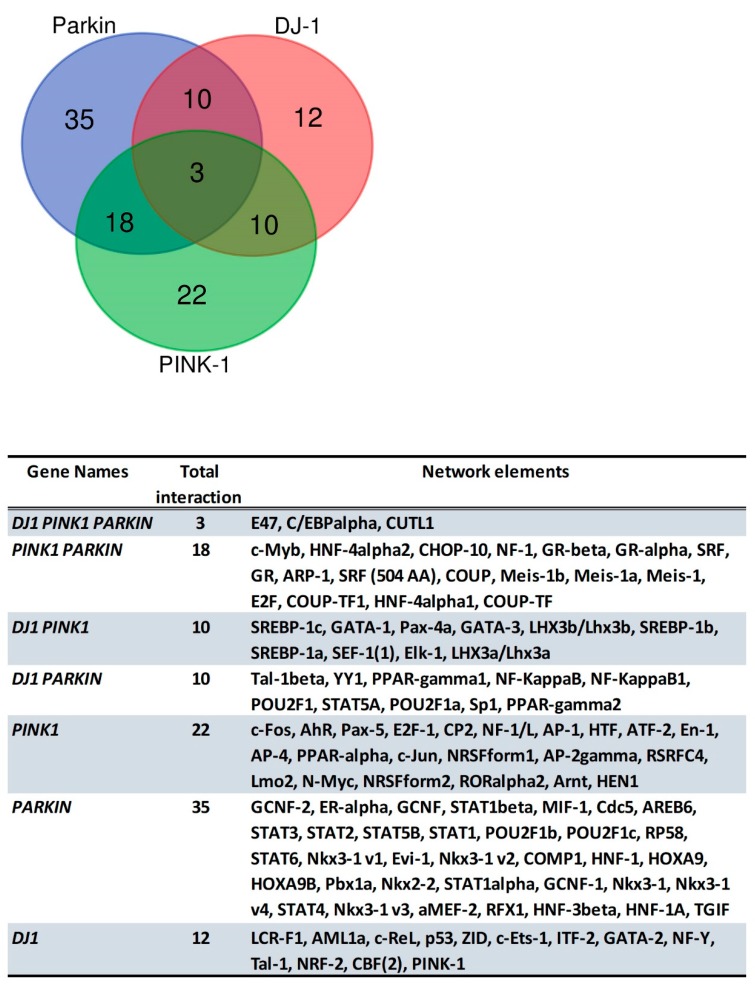
Venn diagram that presents all possible logical relations between the predicted transcription factors for the PINK, PARKIN and PARK7. The diagram was created with the Bioinformatics and Evolutionary Genomics tool.

There are many predicted transcription factors regulating *PINK1, PARK2* (PARKIN) and *PARK7* (DJ-1) gene expression. However, some transcription factors related to cell cycle control are as follows: for *PINK1*, AP-1, and c-Jun; for *PARK7*, SP1, p53, NF-Kappaβ, and NF-Kappaβ1; for *PARK2*, NF-Kappaβ, NF-Kappaβ1, STAT1, STAT1α, STAT1β, STAT14, GR-β, GR-α, GR and E2F (Figure 8). The Venn diagram in Figure 5 presents all possible logical relations between the predicted transcription factors for *PINK1*, *PARK2* (PARKIN) and *PARK7* (DJ-1). The Venn diagram of predicted transcription factor allowed us to recognize three common transcription factors for *PINK1*, *PARK2*, and *PARK7*. These transcription factors are E47, C/EBPα and CUTL1, and may play a role in the interplay between PINK1, PARKIN, and DJ-1 (Figure 8). These transcription factors related to the cell cycle suggests that the interaction among PINK1/PARKIN/DJ-1 network during mitochondrial quality control in cancer biology may be regulated at the transcriptional level. Further analysis, like a systems biology approach, will be helpful in the understanding of the PINK1/PARKIN/DJ-1 network.

## 7. Conclusions

**Figure 9:**
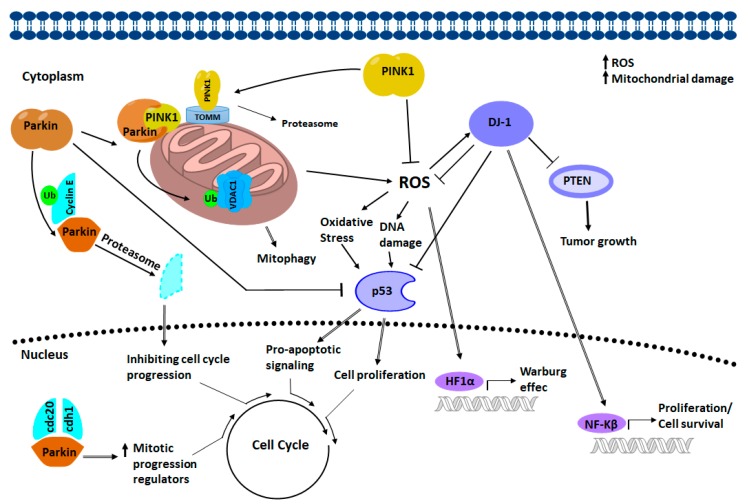
Representation of PARKIN/PINK1/DJ-1 network. The directionality and the nature of the interactions (activation or inhibition) are shown as they are believed to exist in a healthy state. PARKIN interacts with PINK1 to promote the removal of defective mitochondria through the ubiquitination of the voltage-dependent anionic channel 1 (VDAC1), a component of the mitochondrial permeability transition pore that is involved in apoptosis, contributing to limit the damage produced by ROS.

There is an interplay between DJ-1, PINK1, and PARKIN in cancer biology (Figure 9). During late anaphase to G1 phase, PARKIN interacts with the co-activators of APC/C, Cdc20 and Cdh1 thus, forming a complex that increases the expression of regulators of mitotic progression which in turn suppresses the progression of the cell cycle. Also, PARKIN represses cyclin E levels, also through ubiquitination (Ub), by inhibiting the progression of the cell cycle, stopping in G1 phase. PARKIN repressed p53 levels through its interaction with the TP53 promoter region, which could help maintain controlled pro-apoptotic signaling. DJ-1 also represses the expression of TP53, as well as p53 protein levels. The reduction in the expression of PINK1 causes the stabilization of HIF1A through the elevation of ROS. Cancer cells increase aerobic glycolysis through the Warburg effect and produce higher levels of oxidative stress through ROS. Through multiple mechanisms, DJ-1 helps minimize the damage caused by oxidative stress. During cancer development, DJ-1 delivers cytoprotection by independent mechanisms such as NF-κβ and PTEN. DJ-1 activates NF-κβ and inhibits tumor suppressor activity activated by PTEN, leading to cell survival and tumor growth. The mutation or altered expression of these associated genes in cancer interrupts the checkpoints and balances that maintain cellular integrity and that protect against inappropriate cell proliferation. Many of these interruptions converge and drive the cell cycle. The result is determined by whether the cell is capable of mitosis, in which case an over proliferation occurs, or whether the cell is postmitotic, in which case cell death occurs.

The STRING database and Gene Ontology (GO) enrichment analysis were performed to consolidate known and predicted protein-protein association with PINK1, PARKIN, and DJ-1. The enrichment analysis of KEGG pathways showed that the PINK1/PARKIN/DJ-1 network show Parkinson disease as the main feature. Only the protein DJ-1 showed enrichment in prostate cancer and p53 signaling pathway. Some predicted transcription factors regulating *PINK1*, *PARK2* (PARKIN) and *PARK7* (DJ-1) gene expression are related to cell cycle control. We suggest that the interplay among PINK1/PARKIN/DJ-1 network during mitochondrial quality control in cancer biology may occur at the transcriptional level. Further analysis, like a systems biology approach, will be helpful in the understanding of PINK1/PARKIN/DJ-1 network.

## Figures and Tables

**Table 1:** Main equations involved in free radical biochemistry.

Metal-Catalyzed Haber-Weiss Reaction
${\mathrm{Fe}}^{3+}+\left({\mathrm{Cu}}^{2+}\right)\to {\mathrm{Fe}}^{2+}\left({\mathrm{Cu}}^{+}\right)+O_{2}$ ^(1)^
${\mathrm{Fe}}^{3+}+H_{2}O_{2}\to {\;\mathrm{Fe}}^{3+}+{\mathrm{OH}}^{-}+{\mathrm{OH}}^{•}$ ^(2)^
$O_{2}{}^{•-}+H_{2}O_{2}\to O_{2}+{\mathrm{OH}}^{-}+{\mathrm{OH}}^{•}$ ^(3)^
${\;\mathrm{NO}}_{2}{}^{-}\;\mathrm{reductase}\;\mathrm{activity}\;\mathrm{Cco}/{\mathrm{NO}}^{•}$
${\;\mathrm{NO}}_{2}{}^{-}+{\mathrm{Fe}}^{2+}+H^{+}\to {\mathrm{NO}\;}^{•}+{\mathrm{Fe}}^{3+}+{\mathrm{OH}}^{-}$

^(1)^ Reduction of ferric ion to ferrous, ^(2)^ Fenton reaction, ^(3)^ The net reaction. O_2_, Diatomic oxygen; O_2_^•−^, Superoxide; H_2_O_2_, Hydrogen Peroxide, OH^•^, Hydroxyl radical.
